# Blessing or burden? Long‐term maintenance, complications and clinical outcome of intrathecal baclofen pumps†

**DOI:** 10.1111/1744-1633.12308

**Published:** 2018-07-04

**Authors:** David Yuen‐Chung Chan, Steve Sik‐Kwan Chan, Emily Kit‐Ying Chan, Amelia Yikjin Ng, Aaron Chee‐Lun Ying, Ara Cheuk‐Yin Li, Candy Ching‐Pik Chiu, Ning Cheung, Wai‐Kit Mak, David Tin‐Fung Sun, Cannon Xian‐Lun Zhu, Wai‐Sang Poon

**Affiliations:** ^1^ Division of Neurosurgery, Departments of Surgery The Chinese University of Hong Kong Hong Kong; ^2^ Anaesthesia and Intensive Care, Prince of Wales Hospital The Chinese University of Hong Kong Hong Kong

**Keywords:** baclofen, infection, intrathecal, spasticity

## Abstract

**Aim:**

The intrathecal baclofen pump is an effective treatment for spasticity. However, long‐term results have reported patients’ dissatisfaction and perception of disability. Potential causes include a frequent need for baclofen pump refill and risks of complications. The aim of the present study was to evaluate the long‐term maintenance, complications and clinical outcome of intrathecal baclofen pumps.

**Patients and Methods:**

We conducted a 16‐year retrospective cohort study of patients with spasticity treated with an intrathecal baclofen pump at a university hospital from 2000 to 2016. The primary outcome was the rate of infection per puncture for baclofen pump refill. Secondary outcomes included the incidence of other complications, such as running out of baclofen causing symptomatic withdrawal symptoms, pump mechanical failure, pump battery end of life and the need for pump replacement. The clinical outcome was assessed by the Modified Ashworth Scale (mAS).

**Results:**

In total, 340 follow‐up episodes with pump refill procedures were recorded. The average interval between each pump refill was 57.3 days (±15.4 days). The average duration of admission for each pump refill was 4 h and 49 min (from 2 h 23 min to 10 h). There were two events with established infection after puncture for the refill, giving rise to an infection rate per puncture of 0.6 percent (2/340).

For the long‐term clinical outcome, at an average follow‐up period of 7.6 years, the postoperative mAS for spasticity was 2.0 ± 0.756, which was significantly better than the preoperative mAS at 3.75 ± 0.462 (*P* = 0.001).

**Conclusion:**

Long‐term aftercare with baclofen pump refill was safe, with an infection rate of 0.6 per cent per puncture for each refill. Long‐term intrathecal baclofen pump was effective in the treatment of spasticity with persistent significant improvement in the spasticity scale.

## Introduction

The intrathecal baclofen pump is an effective treatment for spasticity.^1^, ^2^, ^3^ However, long‐term results have reported potential patients’ dissatisfaction and perception of disability.^4^ Potential causes include a frequent need for baclofen pump refill and risks of complications.^5^, ^6^, ^7^ The aim of the present study was to evaluate the long‐term maintenance, complications and clinical outcome.

## Methods

**Figure 1:**
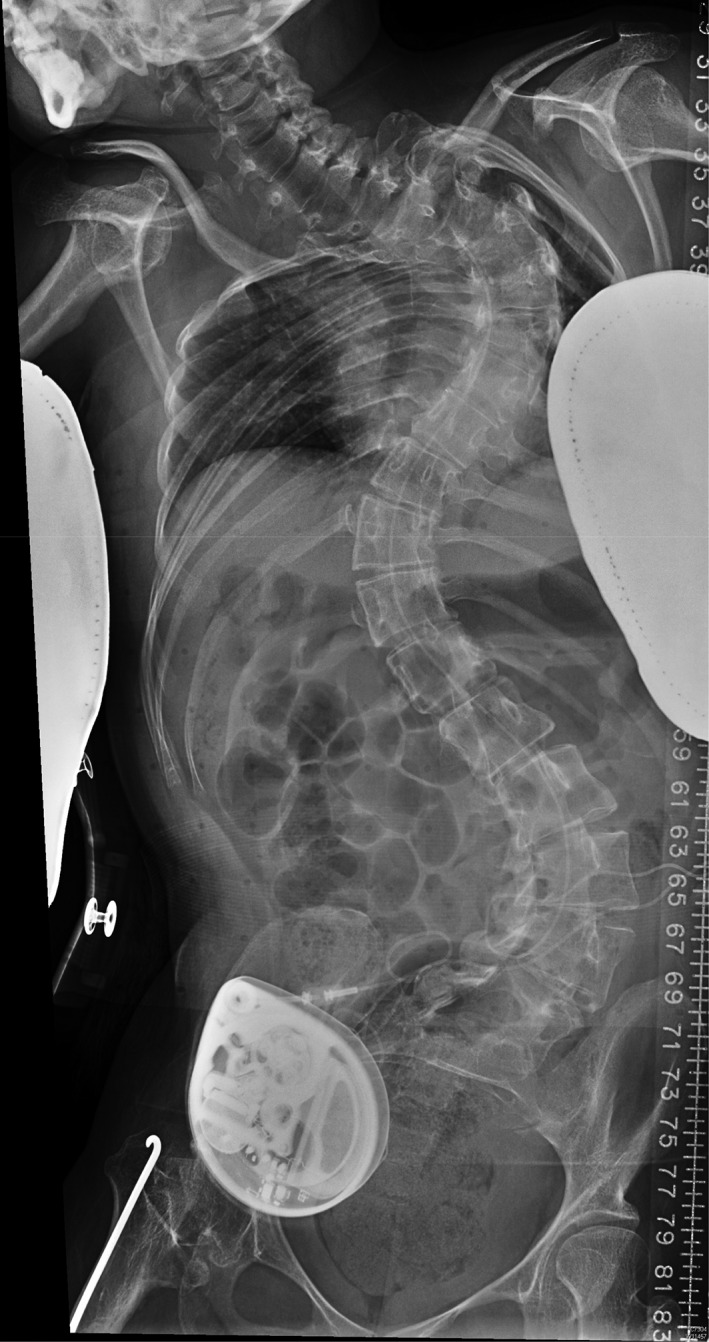
X‐ray spine anterior–posterior view of a patient with severe spasticity treated with an intrathecal baclofen pump. Note the associated severe scoliosis. In view of the distortion, the X‐ray was reviewed for orientation of the pump at the right lower quadrant of the abdomen before needle puncture through the skin and subcutaneous tissue into the pump for refilling of baclofen into the reservoir.

**Figure 2:**
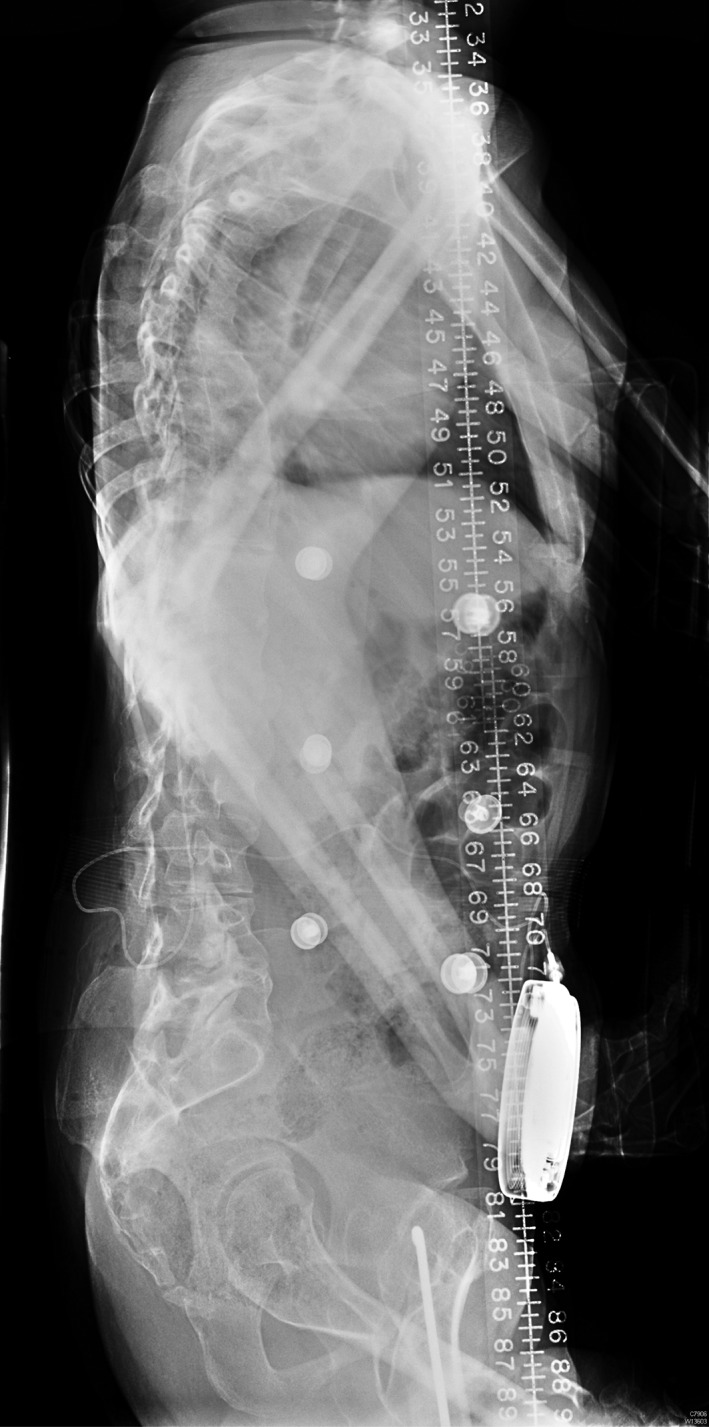
Spine lateral view of the same patient. Entry site of the lumbar puncture was at the L4/5 level, and the subdural catheter was inserted until the tip reached the lower cervical region, with the aim of relieving the muscle tone of the upper limbs, torso and lower limbs.

We conducted a 16‐year retrospective cohort study of patients with spasticity treated with intrathecal baclofen pump at a University Hospital (Prince of Wales Hospital, Hong Kong) from 2000 to 2016. The inclusion criterion was patients treated with implantation of an intrathecal baclofen pump for spasticity during the study period. Exclusion criteria included patients who were lost to follow‐up and those receiving follow‐up treatment at other hospitals. The primary outcome was the rate of infection per puncture for baclofen pump refill. Secondary outcomes included the incidence of other complications, such as running out of baclofen causing symptomatic withdrawal symptoms, pump mechanical failure, pump battery end of life and the need for pump replacement. Clinical outcome was assessed by the Modified Ashworth Scale (mAS). All of the baclofen pumps in this study have a reservoir volume of 20 mL. For each refill, 20 mL intrathecal baclofen with a concentration of 2000 μg/mL was instilled into the pump reservoir, giving rise to a total of 40 000 μg (or 40 mg). The criterion for refill was set for patients to return before the residual volume reached 1 mL. The baclofen pump low‐volume alarm was set at 1 mL for safety reasons, and an elective refill appointment was scheduled to the closest weekday, 1–2 days before the alarm date, as calculated by each patient's baclofen daily usage rate. For our centre, baclofen pump refill was performed at bedside or in the minor procedure room in the day surgical ward. Logistics included prescribing baclofen after admission and then obtaining the medication from the pharmacy before the actual medication injection into the pump. For cases with severe spasticity and distorted anatomy, previous X‐rays of the spine and abdomen were reviewed for orientation before needle injection into the pump (Figs 1, 2). This duration of admission for pump refill in the day ward setting was also analysed as one of the secondary outcomes. Statistical analysis was performed with χ^2^‐test, Fisher's exact test and Student's *t*‐test. Significance was set at 5 per cent, and all statistical analyses were done with SPSS software version 22.0 (IBM, New York, NY, USA).

## Results

In total 340 follow‐up episodes with pump refill procedures were recorded for nine patients. The average follow‐up period was 7.6 years (range: 2–15 years). The average interval between each pump refill was 57.3 days (±15.4 days). The average duration of admission for each pump refill was 4 h and 49 min (from 2 h 23 min to 10 h). There were two events with established infection after puncture for refill, giving rise to an infection rate per puncture of 0.6 percent (2/340).

In total, 10 patients were identified during the study period. Data from one of the patients was missing as she did not come back for follow‐up. She returned to Beijing, China, after the operation for implantation of a baclofen pump and did not have a subsequent follow up or baclofen refill at the Prince of Wales Hospital, Hong Kong. This patient was therefore excluded from the study. The average follow‐up period was 7.6 years (range: 2–15 years).

The average age of the patient at the time of operation was 33.3 years (11–58 years). For the underlying disease pathology, three had spinal cord injury, three had diplegic cerebral palsy, two had intracranial haemorrhage and one had multiple sclerosis.

For the secondary outcome, the incidence of other complications, such as running out of baclofen causing symptomatic withdrawal symptoms, pump mechanical failure, pump battery end of life and the need for pump replacement, were analysed. There was no recorded incidence of running out of baclofen during the study period from 2000. There were four pumps reaching battery end‐of life requiring reoperation for the implantation of new pump after an average usage of 8.6 years (range: 3–13 years). There was one episode of symptomatic withdrawal due to the premature consumption of the pump battery 3 years after implantation of pump. The patient presented with spasm and sweating, which were relieved by diazepam and baclofen. Operation for change of pump with a new model was performed after the patient was stabilized. Intraoperative findings revealed kinking of the old connection tubing, which can be accountable for the excessive battery consumption leading to premature end of life of the pump. These were corrected during the subsequent reoperation, and the patient was stable with good symptomatic relief 11 years postoperatively.

For the clinical outcome, all had severe spasticity with an average mAS of 3.75 ± 0.462 before the operation. Postinfusion trial mAS was 1.625 ± 0.744 (*P* = 0.001). Postoperative mAS upon last follow up was 2.0 ± 0.756 (*P* = 0.001), which was significantly improved.

**Figure 3:**
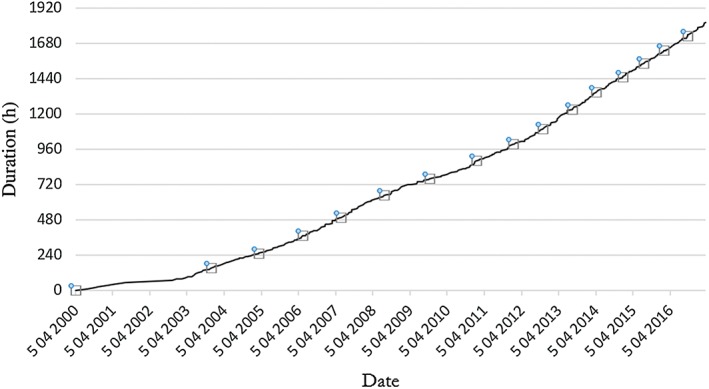
Results showing the cumulative total admission time for the refill of intrathecal baclofen pump at the Prince of Wales Hospital, Hong Kong, from 2000 to 2016. In the early period, from 2000 to 2003, when there were only two patients with intrathecal baclofen pumps, the average interval for a medical staff to refill a baclofen pump was 38.1 days. Subsequently, the slope of the curve increased exponentially as more patients were implanted with intrathecal baclofen pumps. Average interval for a medical staff to refill a pump was 9.97 days in 2016, which was significantly more frequent than 38.1 days in 2000 (*P* = 0.001). [Color figure can be viewed at http://wileyonlinelibrary.com]

With a total of 340 cumulative admission episodes for intrathecal baclofen pump refill at the study centre, we generated a cumulative time of in‐hospital stay for baclofen pump refill from 2000 to 2016, as illustrated in Figure 3. The average dosage of baclofen used was 385 μg/day (range: 129.9–631 μg/day). In the early period, from 2000 to 2003, when there were only two patients with intrathecal baclofen pumps, the average interval for a medical staff to refill a baclofen pump was 38.1 days. Subsequently, the slope of the curve increased exponentially as more patients were implanted with intrathecal baclofen pumps. The average interval for pump refill was 9.97 days in 2016, which was significantly more frequent than 38.1 days in 2000 (*P* = 0.001).

## Discussion

In a randomised, double‐blind, multicentre trial in 1997, Middel *et al*. reported significant improvement in the spasticity of patients with the use of intrathecal baclofen pumps.^1^ However for the same cohort of patients, Zahavi *et al*. identified ‘a small but significant worsening of disability’ and ‘perceived health status’ (pp. 1553–1557).^4^ These were illustrated by a worsening score for the Expanded Disability Status Scale, the Ambulatory Index and the Incapacity Status Scale.^4^ In fact, McCormick *et al*. showed that, although long‐term use of intrathecal baclofen had a significant reduction in spasticity as compared to oral baclofen, there were no significant differences in pain, sleep, fatigue and quality of life.^8^

Our study specifically analysed the average time spent for each patient to refill the baclofen pump in a hospital‐based setting. We hope this study can fill the gap in the literature, as this information was not readily available during counselling for patients before the operation. As for the average interval between each refill, we understood that the medication dosage requirement varied between individual patients with different underlying pathologies. The aim of the present study was to provide an objective figure with a reasonable range for patients and their relatives to comprehend the gravity of the subsequent treatment requirement during the long‐term aftercare of the intrathecal pump. The causation relationship between patients’ perceived disability status and the frequency or duration of pump refill remained to be elucidated.

The clinical outcome in the present study in terms of degree of improvement in mAS was comparable with the published data in the literature: The preoperative average mAS ranged from 2.51 to 2.82, which improved to 0.91–1.51 at 3 months to 5 years postoperatively (*P* < 0.05).^1^, ^4^ In contrast, the patients in our study had more severe preoperative spasticity, with an average mAS of 3.75 ± 0.462 preoperatively. With a comparable degree of improvement, the average postoperative mAS was 2.0 ± 0.756.

In general, the infection rate in published data ranges from 2.63 to 16.67 per cent per baclofen pump implanted. In the present study, we specifically looked into the infection rate per puncture, as it was not reported in the literature previously; our study generated an infection rate of 0.6 per cent per puncture.

Al‐Shaar *et al*. reported on intrathecal baclofen pump compliance and found that only 74.1 per cent of patients in their study continued to use the intrathecal baclofen pump at 2‐years’ postimplantation.^9^ There is still a considerable gap in the number of intrathecal baclofen pumps implanted within different regions.^10^ Potential alternative options should be considered, such as ablative spinal procedures.^11^ A benefit–risk study^12^ and a cost‐effectiveness study^13^ supported the use of intrathecal baclofen pumps. Saulino *et al*. advocated that the best practice for intrathecal baclofen pumps was patient selection.^14^

The limitation of the present study was based on its retrospective nature. However, we were able to collect data on 340 episodes of hospital admissions, which was adequate to generate reasonable results for the average duration and interval for intrathecal baclofen pump refill.

**Figure 4:**
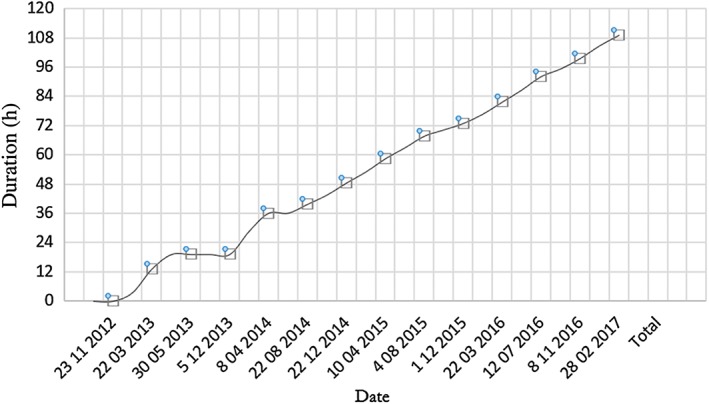
Results showing the cumulative time for a 20‐year‐old gentleman with cerebral palsy treated with intrathecal baclofen pump since 15 years of age. Note that the initial duration of in‐hospital admission time fluctuated due to dosage titration. Subsequently, the slope of the curve was static with stable intrathecal baclofen dosage requirement. With an average baclofen pump refill interval of 62 days and an average admission duration of 4 h and 57 min for each refill, he had accumulated more than 100 h postoperative hospitalization within 4 years. [Color figure can be viewed at http://wileyonlinelibrary.com]

One of the patients in our study was a 20‐year‐old gentleman with cerebral palsy treated with intrathecal baclofen pump since he was 15 years old. The cumulative time of in‐hospital stay for baclofen pump refill is illustrated in Figure 4, in which the initial admission time fluctuated due to dosage titration is noted. Subsequently, the slope of the curve was static with stable intrathecal baclofen dosage requirement. With an average baclofen pump refill interval of 62 days and an average admission duration of 4 h and 57 min for each refill, he had accumulated >100 h of postoperative hospitalization within 4 years. This potentially increased burden and resources for medical staff and the hospital, as the patient required frequent attendance for baclofen pump refill. However, the refill process itself usually takes approximately 15–20 min, in which 2 h and 23 min to 10 h of ‘admission time’ is largely due to waiting. The logistics of baclofen pump refill varies globally. At some centres, baclofen pump refill is performed in the operating theatre as an elective surgery. This setting is cleaner, but it is associated with higher costs. At our centre, it was performed at the bedside or in the minor procedure room in the day surgical ward. It is potentially more efficient, but the aseptic environment is less stringent. The present study served to generate new data and fill the knowledge gap on the infection rate for this procedure to be performed in a day‐ward setting, which is very low. The role of an outpatient‐based baclofen pump refill clinic might be a potential solution, and further study is needed to validate this.

In conclusion, long‐term aftercare with baclofen pump refill was safe, with an infection rate of 0.6 per cent per puncture for each refill. Long‐term intrathecal baclofen pump was effective in the treatment of spasticity, with persistent significant improvement in the spasticity scale. Long‐term aftercare with baclofen pump refill required an average interval of 57.3 days (±15.4 days) and an average duration of admission of 4 h and 49 min (2 h and 23 min to 10 h) for each refill.

## Declaration of conflict of interest

The authors report no conflicts of interest.
